# A Smartphone-Based Timed Up and Go Test Self-Assessment for Older Adults: Validity and Reliability Study

**DOI:** 10.2196/67322

**Published:** 2025-03-21

**Authors:** Melissa Johanna Böttinger, Sabato Mellone, Jochen Klenk, Carl-Philipp Jansen, Marios Stefanakis, Elena Litz, Anastasia Bredenbrock, Jan-Philipp Fischer, Jürgen M Bauer, Clemens Becker, Katharina Gordt-Oesterwind

**Affiliations:** 1 Geriatric Center, Medical Faculty Heidelberg, Heidelberg University, Bergheimer Str. 20, Heidelberg, 69115, Germany, 49 6221 548146; 2 Network Aging Research, Heidelberg University, Heidelberg, Germany; 3 Department of Electrical, Electronic and Information Engineering, University of Bologna, Bologna, Italy; 4 Institute of Epidemiology and Medical Biometry, Ulm University, Ulm, Germany; 5 Study Center Stuttgart, IB University of Health and Social Sciences, Stuttgart, Germany; 6 Department of Clinical Gerontology and Geriatric Rehabilitation, Robert Bosch Hospital, Stuttgart, Germany; 7 TSG ResearchLab gGmbH, Zuzenhausen, Germany; 8 Institute of Sports and Sports Sciences, Heidelberg University, Heidelberg, Germany

**Keywords:** timed up and go test, self-assessment, instrumented assessment, technology-based assess-ment, physical capacity, mobility, aged, mobile applications, smartphone, diagnostic self evaluation

## Abstract

**Background:**

The Timed Up and Go test (TUG) is recommended as an evidence-based tool for measuring physical capacity. Instrumented TUG (iTUG) approaches expand classical supervised clinical applications offering the potential of self-assessment for older adults.

**Objective:**

This study aimed to evaluate the concurrent validity and test-retest reliability of a smartphone-based TUG self-assessment “up&go app.”

**Methods:**

A total of 52 community-dwelling older adults (>67 years old) were recruited. A validated and medically certified system attached with a belt at the lower back was used as a reference system to validate the “up&go app” algorithm. The participants repeated the TUG 5 times wearing, a smartphone with the “up&go app” in their front trouser pocket and an inertial sensor to test the concurrent validity. A subsample of 37 participants repeated the “up&go app” measurement 2 weeks later to examine the test-retest reliability.

**Results:**

The correlation between the “up&go app” and the reference measurement was *r*=0.99 for the total test duration and *r*=0.97 for the 5 single repetitions. Agreement between the 5 repetitions was intraclass correlation coefficient (ICC)=0.9 (0.84‐0.94). Leaving out the first repetition, the agreement was ICC=0.95 (0.92‐0.97). Test-retest agreement had an ICC=0.79 (0.53‐0.9).

**Conclusions:**

The duration of 5 repetitions of the TUG test, measured with the pocket-worn “up&go app,” was very consistent with the results of a lower-back sensor system, indicating excellent concurrent validity. Participants walked slower in the first round than in the other 4 repetitions within a test run. Test-retest reliability was also excellent. The “up&go app” provides a useful smartphone-based approach to measure 5 repetitions of the TUG. The app could be used by older adults as a self-screening and monitoring tool of physical capacity at home and thereby help to early identify functional limitations and take interventions when necessary.

## Introduction

The baby boomer generation embraces smartphone technology [1]. This particular cohort is willing and capable of using mobile health applications especially when they are designed in a user-centered way and increase the likelihood of a self-determined future [2-4]. Good examples are the use of fitness trackers, heart rate monitoring, and glucose monitoring [5].

The World Health Organization (WHO) and United Nations (UN) member states have declared the current decade as the UN “Decade of Healthy Ageing” (2021‐2030) to improve the lives of older people, their families, and the communities in which they live [6-8]. Early identification of health risks and functional limitations in older adults is crucial to enable timely preventive measures and promote the maintenance of independence [9]. A recent systematic review was conducted to guide WHO recommendations and to establish a “standard” for evidence-based assessment of physical capacity [10]. It recommends the Timed Up and Go Test (TUG), in addition to the 30-Second Chair Rise Test, due to the high quality evidence for its sufficiently valid and reliable measurement of physical capacity in community-dwelling older adults [10,11]. The standard TUG captures the time recorded by a trained test administrator using a stopwatch. The test person rises from a chair, walks 3 meters at their usual pace, turns around, walks back, and sits down again. Longer test durations indicate limitations in physical capacity, particularly in strength and balance [10-12] and predict hospitalization and associated functional decline in older adults [13]. The need for and lack of a digital version of the TUG has been addressed during the meetings of the WHO Locomotor Capacity Working Group. This is even more relevant for persons living remotely or in low and middle-income countries where smartphone technology is often available but health care professionals are often lacking [2].

Wearable technologies offer opportunities to lower the barrier for integrating functional tests like the TUG into the assessment and clinical management of older people [14,15]. Such an instrumented Timed Up and Go Test (iTUG) not only enables standardized and digitized measurement in supervised settings [16-18] but also opens an option for low-cost self-screening and monitoring in people’s everyday lives [19,20]. In clinical studies, camera-based systems, inertial sensors or smartphones that are attached to the back (eg, with a belt) are most commonly used to perform the iTUG [21]. For self-assessment purposes, however, an approach using common technologies and a convenient placement on the body would be more feasible, without the need for additional materials or training. Therefore, the “up&go” iTUG was developed as a self-test for older adults. The prototype of the “up&go” app emerged from several EU projects [17-19,22,23] and a cocreation process with older adults [24]. It incorporates an innovative approach: placing a smartphone in the user’s front trouser pocket during 5 repetitions of the test. An unsupervised smartphone-based iTUG could fill a gap in the landscape of mobility assessment methods that is still dominated by patient-reported outcome measures and supervised assessments conducted in laboratory or clinical settings [25]. Digital self-assessments could support older adults to take up an active role in risk screening or monitoring, and to early identify changes in their mobility. If necessary, this would allow them to initiate further medical diagnostics, and to take primary or secondary preventive measures in a timely manner. Thus, the motivation for this study is to examine if the “up&go” self-assessment app fulfils the criteria of validity and reliability to be considered trustworthy and to serve as a credible tool for implementation in future iTUG studies and in the self-management of older people.

Hence, this study aims to examine the concurrent validity and test-retest reliability of the “up&go” smartphone-based TUG self-assessment for older adults. The measures considered are the total TUG test duration and the duration of the 5 individual repetitions. This study corresponds to an analytical validation according to the V3 framework [26].

The objectives of this study are to analyze the concurrent validity of the algorithm used in the “up&go app” against a validated and medically certified sensor-based system and to determine the test-retest reliability of the “up&go app” algorithm in the home environment of older adults.

## Methods

### Participant Recruitment

The findings are reported following the guidelines for reporting reliability and agreement studies [27]. Participant were recruited from the SMART-AGE randomized controlled trial conducted at Heidelberg University, Germany [28]. Participants were included in the SMART-AGE study if they were 67 years or older, lived in the community, had basic knowledge of using PCs or tablets and of the German language. Exclusion criteria were severe medical conditions (ie, heart failure with shortness of breath at rest, cardiac arrhythmia with dizziness, Parkinson disease with use of a walker or wheelchair, cancer with chemotherapy or radiotherapy, chronic lung disease with oxygen therapy, and planned major medical procedure with inpatient hospitalization within the next 3 months), severe visual or hearing impairment, and severe cognitive impairment.

Data for the sample description were extracted from the SMART-AGE initial assessment data: age, sex, BMI, citizenship, living situation, employment, WHO Quality of Life Scale score [29], Satisfaction With Life Scale score [30], fall history, Groll Functional Comorbidity Index [31], Trail Making Test A and B score [32], Six-item Cognitive Impairment Test score [33], Short Falls Efficacy Scale International score [34], 4-Meter Walk Test score [35], 30-second Chair Rise Test score [36], and stopwatch-measured Timed-Up and Go Test score [11].

### Ethical Considerations

The SMART-AGE study and the amended protocol for this sub study were approved by Heidelberg University Medical Faculty’s Ethical Committee (S-672/2022). All participants provided informed signed consent before participating.

### Data Collection and Processing

The data collection was performed during a home visit 3 to 4 months after the SMART-AGE study’s baseline assessment. A total of 3 pretrained assessors with a professional background in psychology (AB and EL) or physiotherapy (MJB) conducted the home visits following a standardized manual.

### “Up&go App”: Smartphone-Based Timed Up and Go Test

Previous versions of the “up&go app” algorithm have been developed and validated within the 2 European Commission funded projects, Farseeing [17,22] and PreventIT [18,19,23]. The “up&go app” investigated in this study [37] is a further development of the previous versions. It is based on the results of a study examining the usability of the previous Norwegian version of the PreventIT project [19] and a cocreation study with older adults [24].

**Figure 1.**
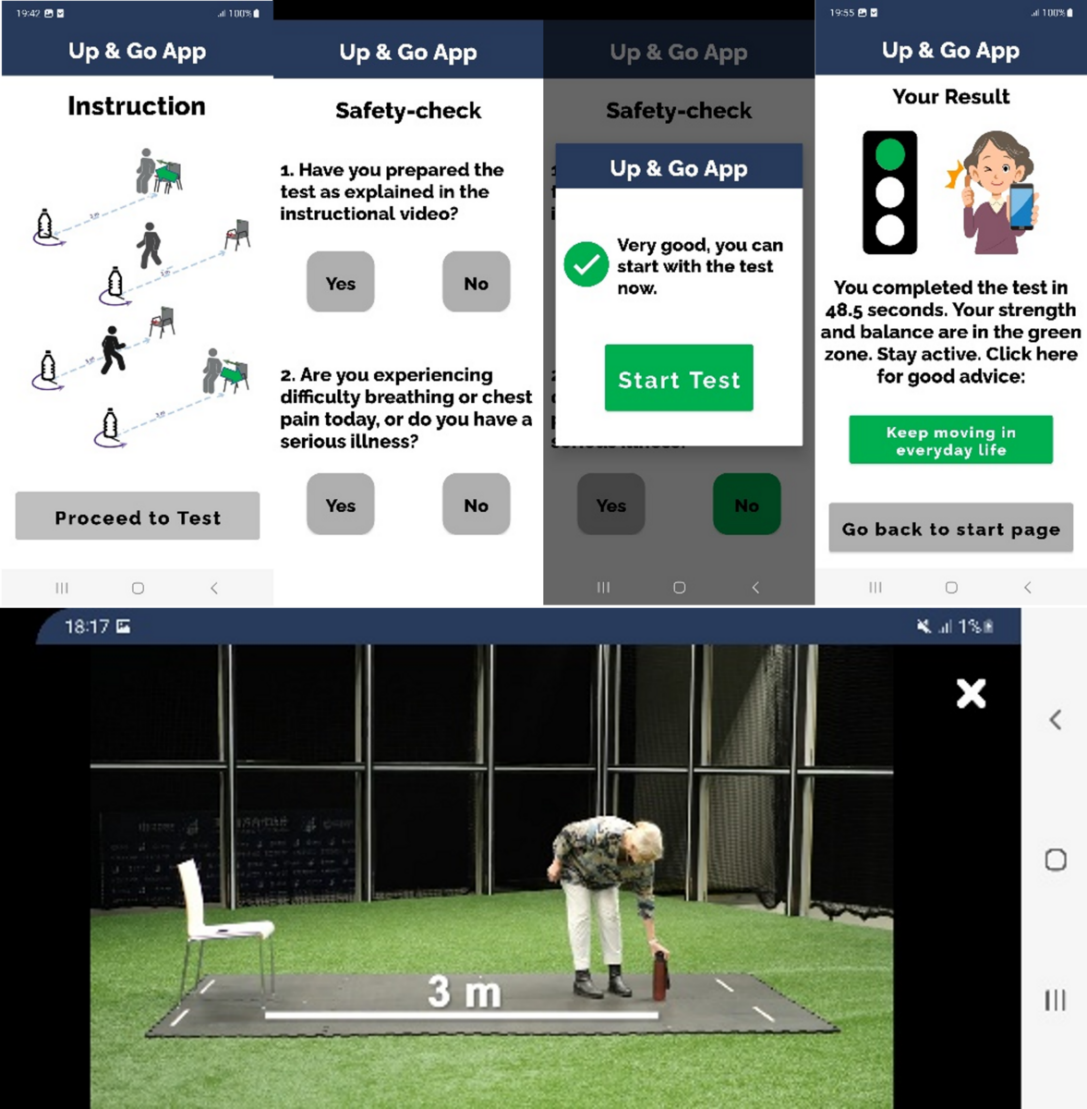
Screenshots from the “up&go app".

In order to run the TUG test within the “up&go app,” the user is invited to watch and read the test instructions, prepare the test setting (chair, 3 m distance, marker), answer 2 safety questions, press the “start test” button, place the smartphone in one of the front trouser pockets and sit down to begin with the first round of the test at usual gait speed (Figure 1). The app includes an algorithm processing real-time accelerometer data to detect the body movements to guide the user through 5 consecutive repetitions of the TUG via audio prompts (eg, “Please put the phone in your front trouser pocket and sit down”). After each run there is a pause of about 3 seconds in which the algorithm detects the position change, and the new audio announcement is played (eg, “This was the third run. Please wait for the next start signal”). The embedded sensors in the smartphone collect data during the test.

After the test, the algorithm automatically postprocesses accelerometer data and computes the total time needed to complete 5 repetitions, excluding pauses between runs. The total time is shown on the smartphone screen in seconds together with a traffic light (Figure 1, green=<60 s, yellow 60‐90 s, red>90 s) and an according recommendation for action (eg, green light: recommendation to stay active, yellow light: recommendation for supervised training, and red light: recommendation to seek medical advice). The color coding thresholds are based on the cut-off value of 12 seconds for 1 repetition, respectively 60 seconds for 5 repetitions, to indicate normal versus below normal mobility in community-dwelling older adults [13,38].

### Concurrent Validity

In this study, the “mTUG” system (“mTUG” medical device, mHealth Technologies) was used as a reference measure to validate the “up&go app” algorithm. “mTUG” is a sensor-based system that has been validated in a population of older adults [17] and is certified as a medical device. The system consists of an inertial sensor (mHealth Technologies triaxial accelerometer range±2 g, triaxial gyroscope range±250°/sec, sampling rate: 100 Hz) connected by Bluetooth to an assessor smartphone (Samsung Galaxy S10e, Android 5.0.1) including a customized app (mHealth Technologies) allowing the assessor to manually start and stop the sensor measurement.

**Figure 2.**
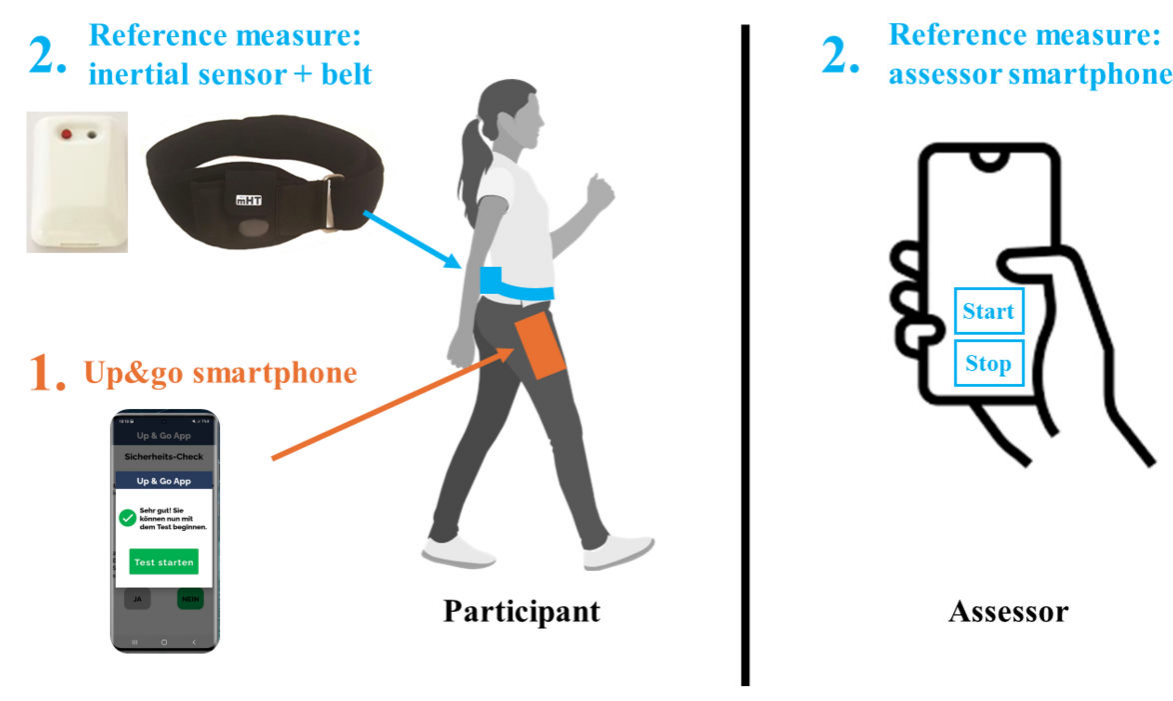
: Simultaneous measurement with (1) the “up&go app” and (2) the reference measure.

The TUG was repeated 5 times with participants wearing the 2 measurement systems simultaneously (Figure 2), smartphone with “up&go app” in their right front trouser pocket and an inertial sensor (mHealth Technologies) attached to an elastic belt worn on the lower back. A previous study with hospitalized hip fracture patients found that the performance of three TUG trials is recommended to achieve performance stability [39]. In this study, 5 repetitions were performed to take into account more variability in the performance of older adults conducting the test at home.

Participants were familiar with the TUG procedure as the conventional stopwatch TUG [11] had been performed beforehand as part of the baseline motor assessments within the SMART-AGE study. As the feasibility of the “up&go app” self-assessment was not part of this study, test preparation, viewing of instructional videos by participants were omitted for data collection. The test procedure was explained to the study partner (5 repetitions, usual walking pace, and follow audio instructions). The assessor prepared the TUG test setting, mounted the sensor in the belt on the participant’s lower back and started the test on the “mTUG” assessor smartphone. Then, the assessor started the test on the “up&go” smartphone and handed it over to the participant. The participant then placed it in the right front trouser pocket and performed the 5 repetitions of the TUG test, following the audio instructions of the “up&go app.”

The “up&go app” does not store any data on the user’s smartphone to guarantee data confidentiality. We therefore developed an adapted version for this study, allowing the app to store all raw data collected from the accelerometer and gyroscope embedded in a Samsung Galaxy S21 smartphone (Android 5.0.1, triaxial accelerometer range±8 g, triaxial gyroscope range±1000°/sec, sampling rate 500 Hz) on the smartphone memory. This allowed postprocessing of the raw data and analysis of not only the total test duration, but also the duration of each of the 5 repetitions (rep1, rep2, rep3, rep4, and rep5).

Within the “mTUG” system, raw data was automatically stored on the assessor smartphone’s memory and processed by a validated algorithm [17].

### Test-Retest Reliability

To investigate the test-retest reliability of the “up&go app” algorithm, we repeated the “up&go” measurement at an additional home visit within 2 weeks after the first measurement, following the same procedure as described above.

Participants were asked to participate in the retest if they lived <20 km from the study center to reduce the burden of assessor time.

### Statistical Analysis

The sample size for the validation part of the study was calculated using R (version 2023.09.1, R Foundation for Statistical Computing, package “pwr”). A strong correlation of ≥0.75 was assumed. With a power of 95% and a statistical significance level of *α*=.05, ≥45 participants are required. The sample size for the test-retest part of the study was calculated using R (version 2023.09.1, package “ICC.sample.size”). An intraclass correlation coefficient (ICC) of ≥0.75 was assumed. With power=95% and *α*=.05, ≥37 participants were required for 2 measurements (first measurement vs retest). Assuming a 15% dropout rate, 52 participants were recruited for the study. From this sample, we consecutively recruited participants for the retest measurement until a subsample size of at least 37 participants was reached to examine the test-retest reliability of the “up&go app” algorithm. The distribution of the data was checked before analyses (Shapiro-Wilk test). All data fulfilled the normal distribution assumption. For all statistical tests, *α*=.05 was used as the threshold for statistical significance. Pearson correlation coefficients were calculated to examine the association between the systems (“up&go app” and reference measure) regarding the total duration needed to complete the test as well as the duration of the 5 single repetitions. Correlation coefficients of 0‐0.19 are interpreted as very weak, 0.2‐0.39 as weak, 0.4‐0.59 as moderate, 0.6‐0.79 as strong, and 0.8‐1 as very strong [40]. For graphical description of the agreement between the 2 systems (“up&go app” and reference measure), Bland-Altman plots were used [41], including the lower and upper limits of agreement (LLoA and ULoA). From the data collected during the first measurement (“up&go app” and reference measure), ICC 3,1 were calculated to analyze the agreement between the 5 repetitions. Test-retest reliability of the “up&go app” was evaluated using ICC [1,2,42]. An ICC value<0.4 indicates poor reliability, an ICC value between ≥0.4 and <0.75 fair to good reliability and an ICC value ≥0.75 indicates excellent reliability [43]. It was assumed that the association between “up&go app” and the reference measure is very strong (*r*>0.8) and the agreement between “up&go” and the reference measure is good (Bland-Altman plot). Furthermore, we assumed that the test-retest reliability of the “up&go” test meets excellent levels (ICC≥0.75). Statistical analyses were computed using statistical software R (version 2023.09.1) and MATLAB (version R2022b, MathWorks).

## Results

### Participant Data

A total of 52 community-dwelling older adults aged between 66 and 88 years (mean 73.6, SD 5.4) were measured, with a similar distribution of men (25/52, 48%) and women (27/52, 52%) and a mean BMI of 27.1 kg/m^2^ (SD 4.1). Of those who provided the data, all were retired, almost all were German citizens (49/52, 98%) and one third (16/52, 33%) lived alone. Participants indicated an average of 2.3 (SD 1.7) comorbidities according to the Groll Index. Of these, degenerative disc disease (17/52), visual impairment (13/52), depression (11/52), and osteoarthritis (11/52) were reported most frequently. Cognition was normal (<7 errors on the Six-item Cognitive Impairment Test) and executive functions (Trail Making Test A and B) were in accordance with normative data of this age group [44]. The average duration of the TUG measured with the stopwatch in the SMART-AGE initial assessment was 9.7 (SD 1.7) seconds. None of the study participants used a walking aid during the TUG. Table 1 provides a detailed description of the sample.

**Table 1.** Characteristics of the study sample.

Data from the SMART-AGE initial assessment^a^		Values	Available sample
Age (years), mean (SD)		73.6 (5.4)	52
Female sex, n (%)		27 (52)	52
BMI (kg/m^2^), mean (SD)		27.1 (4.1)	52
German citizenship, n (%)		49 (98)	50
Living alone, n (%)		16 (33)	49
Retired, n (%)		50 (100)	50
Stopwatch-measured TUG (s), mean (SD)		9.7 (1.7)	52
**4-meter walk test**, **mean (SD)**			
	Duration (s)	4.2 (0.9)	52
	Gait speed (m/s)	1.0 (0.2)	52
**30-Second chair rise test, mean (SD)**			
	Number of repetitions	12.3 (3.3)	52
**Short Falls Efficacy Scale International, mean (SD)**			
	Score	8.2 (2.2)	49
Groll Functional Comorbidity Index (score), mean (SD)		2.3 (1.7)	48
Fall history (“Yes, I fell in the last 12 months”)^b^, n (%)		19 (37)	51
**Trail making test, mean (SD)**			
	Test duration A (s),	45.2 (16.6)	52
	Test duration B (s)	99 (50.3)	52
	Ratio (B/A)	2.3 (1.1)	52
**Six-item cognitive impairment test** ^**c**^ **, mean (SD)**			
	Score	1.0 (1.5)	52
**Satisfaction with life scale, mean (SD)**			
	Score	5.0 (1.2)	49
**WHO Quality of Life Scale, mean (SD)**			
	Physical domain (score)	77.3 (13.1)	49
	Psychological domain (score)	72.4 (10.3)	49
	Social relationships domain (score)	65.3 (13.6)	49
	Environment domain (score)	79.8 (10.1)	49

a The initial SMART-AGE assessment took place 3-4 months before the data collection for this study.

b Fall history data was obtained one month after the initial SMART-AGE assessment.

c Participants with >7 error points were excluded from the SMART-AGE study due to a suspected dementia disorder.

### Concurrent Validity

In total, 35 (67.3%) participants completed the test successfully on the first attempt. Due to technical problems (eg, smartphone shifted in trouser pocket), 12 (23.1%) participants had to repeat the test a second time, 4 (7.7%) participants a third time, and 1 (1.9%) participant a fourth time. After an unsuccessful attempt, participants always took a break of at least 5 minutes. As the app only saves data from complete test runs, failed attempts were not included.

On average, participants needed 52.4 (SD 7.8) seconds to complete 5 repetitions of the TUG, as measured by the “up&go app”. Table 2 shows the results for the total duration of the test and the duration of the 5 repetitions as measured by the “up&go app” and the reference measure.

**Figure 3.**
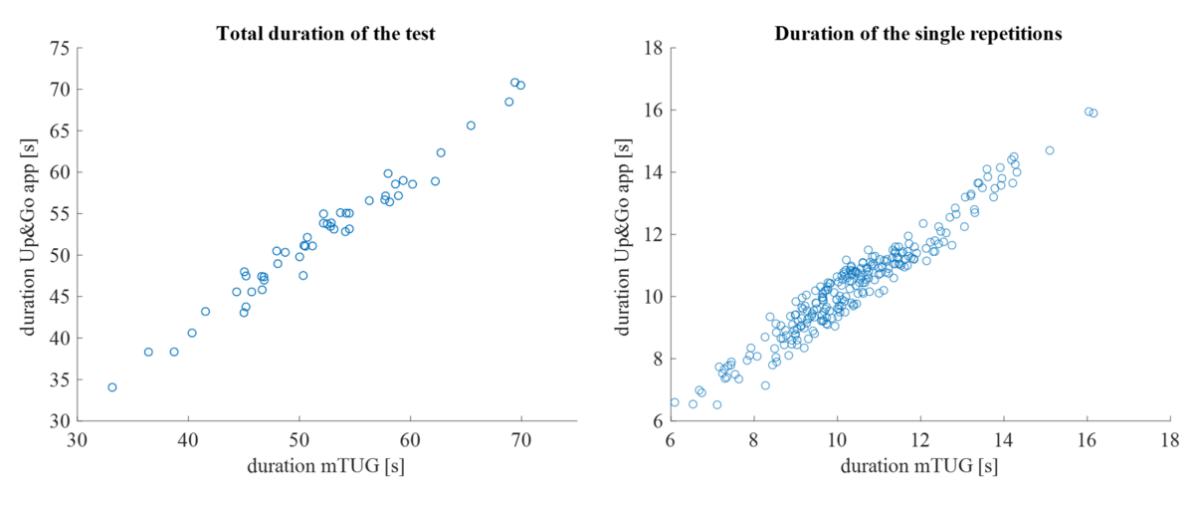
Correlation analysis between “up&go app” and reference measure: total duration and duration of the single repetitions. TUG: Timed Up and Go Test.

Data from all 52 participants were available for the concurrent validity analysis (“up&go app” vs reference measure). The Pearson correlation coefficient (*r*) for the total duration was 0.99 (95% CI 0.98‐0.99) and that for the durations of the 5 single repetitions was 0.97 (95% CI 0.96-.97; Figure 3).

**Table 2.** Total test duration and duration of the 5 repetitions measured by the “up&go app” and reference measure.

Duration	“up&go app”, mean (SD)		Reference measure, mean (SD)	
Total duration of the test (s)	52.38 (7.8)		52.31 (8.11)	
First repetition (s)	10.99 (2.1)		11.11 (2.17)	
Second repetition (s)	10.33 (1.54)		10.39 (1.61)	
Third repetition (s)	10.25 (1.57)		10.34 (1.64)	
Fourth repetition (s)	10.21 (1.52)		10.2 (1.52)	
Fifth repetition (s)	10.11 (1.46)		10.26 (1.6)	

**Figure 4.**
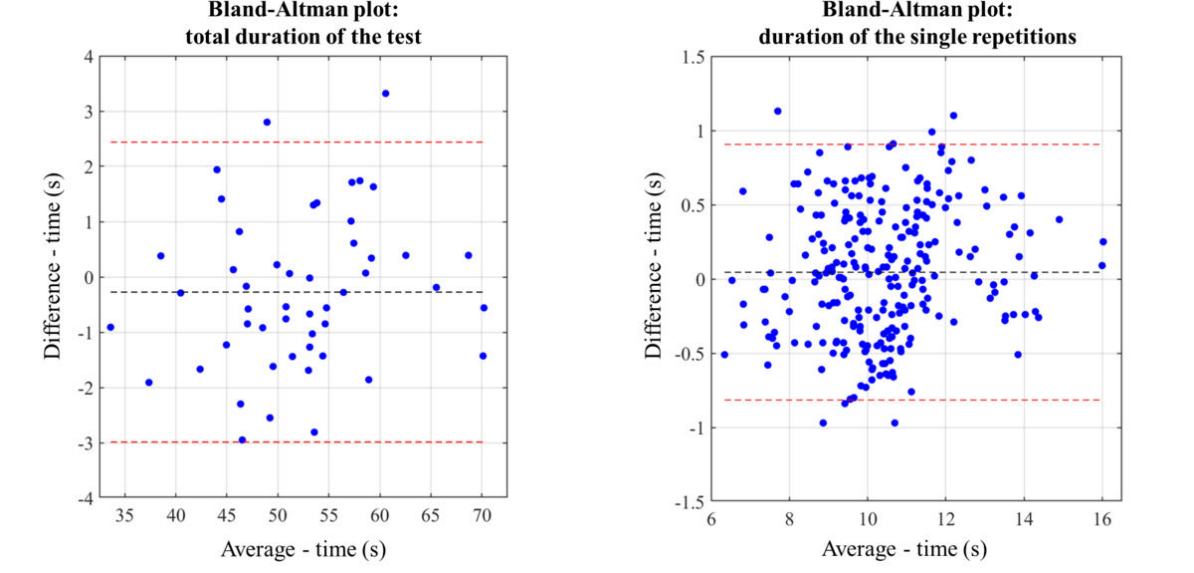
Statistical agreement between the “up&go app” and the reference measure—Bland-Altman plot.

The agreement between the total duration measured by the “up&go app” and the reference measure is shown in Figure 4 (Bland-Altman plots). According to Bland-Altman analysis, the mean difference was −0.27 for the total duration of the test and −0.05 for the duration of the single repetitions. The difference in the total duration of the test was between −2.99 seconds (LLoA) and −2.44 seconds (ULoA) and the difference in the duration of a single repetition was within −0.82 seconds (LLoA) and −0.91 seconds (ULoA) with respect to the estimate of the reference measure.

Agreement between all “up&go app” repetitions was ICC(3,1)=0.9 (95% CI 0.84‐0.94) and ICC(3,1)=0.87 (95% CI 0.80‐0.93) between all repetitions measured by the reference measure. Leaving out the first repetition, the agreement of repetitions 2‐5 was ICC(3,1)=0.95 (95% CI 0.92‐0.97) for the “up&go app” and ICC(3,1)=0.93 (95% CI 0.89‐0.96) for the reference measure.

### Test-Retest Reliability

Of the 52 participants, 37 were measured twice, and 1 participant was excluded due to noncompliance with the retest instruction to walk at habitual gait speed. Retests were performed on average 6 (SD 2.6) days after the first measurement. At the “up&go app” retest measurement, participants needed on average 48.6 (SD 8.5) seconds to complete the 5 repetitions. The ICC between the total duration during the first home visit and during the retest was ICC(2,1)=0.79 (95% CI 0.53‐0.9).

## Discussion

### Principal Findings

To enable self-assessment for older people in their own homes, the “up&go app” was developed as a pocket-worn approach with 5 repetitions of the TUG. This study aimed to investigate the concurrent validity and test-retest reliability of the “up&go app,” which records and processes data from sensors embedded in a smartphone that is placed in the front pocket of the trousers. The results show excellent agreement between the “up&go” data and the comparator system, a previously validated medically certified lower-back inertial sensor system. Overall, the correlation of the 5 single repetitions was very strong. The results indicate that participants did not seem to walk at the same speed in each of the 5 test rounds, that is, they tended to walk slower during the first round. This trend probably shows a learning or accustoming effect. Similar observations have been made in previous studies [39,45] where participants started rather slowly and more carefully during the first attempt of motor performance tests to follow the instructions precisely and to avoid errors [45]. This first round may therefore be comparable to a “trial run,” which is often performed in assessments before the actual measurement. Performing several repetitions of the test provides the opportunity to observe any changes in walking speed during the test and, if necessary, to interpret it clinically regarding physical capacity. For example, in more frail populations, a significant reduction in walking speed during the test could indicate fatigue. Furthermore, this approach might achieve a better approximation toward the “real” usual walking speed compared with measuring only 1 or 2 rounds. Using the app to monitor differences in the total test duration across several measurements would be a clinically relevant use case. But what would be considered a “relevant change”? Minimal important differences (MID) of >2 seconds for 1 TUG repetition were reported in studies observing populations with age-associated disorders and TUG baseline values around 20 seconds [46,47]. In another study observing older adults with hip osteoarthritis and a TUG baseline duration of 7 seconds, a sample that is comparable with this study, the MID is significantly lower with, 0.8 seconds [48]. This suggests that ceiling effects make it more difficult to screen for relevant changes in more robust individuals. The “up&go app,” however, should not only aim to screen for subjectively noticeable changes, but also for changes falling below the abovementioned MID thresholds. Looking at the Bland-Altman plots, a clinically relevant change could be assumed if the measured change is outside the limits of agreement. The limits identified in this study were about ±3 seconds (LLoA, ULoA) for the total duration of 5 repetitions and about ±1 second (LLoA, ULoA) for a single round. Especially in relatively fit, prefrail older adults, a 5-repetition approach could be more sensitive to capture small changes and clinically more useful for early identification of declining physical capacity.

The test-retest reliability analysis showed excellent results. Looking at the average total test duration, we observed a shorter total test duration in the retest. On average, retests took place 6 days after the initial measurement, which makes an improved TUG test performance through training effects unlikely. A similar observation was described elsewhere [45]. A learning effect could account for the shorter duration of the retest. Since participants were already familiar with the test from the first measurement, they may have been more confident and faster during the retest. Other iTUG systems measuring at the height of the navel, (ICC=0.97) [49] and the lower back (ICC=0.9‐0.96) [50] show slightly higher test-retest reliability values. This is to be expected, as the “up&go app” pocket position, specifically selected for the self-assessment purpose, is located more distant from the center of mass. Another reason could be that individuals wore different clothes with different types of pockets; these pockets may be tight or loose, which aggravates individual differences.

For future monitoring purposes, machine learning could be implemented to enable an in-app plausibility check. This might be a helpful feature to detect implausible changes in test duration (eg, >20% compared with previous test performances very recently).

### Limitations

The sample was predominantly White, and participants were relatively fit, as demonstrated by the TUG performance. The average time needed to complete one repetition (9.7, SD 1.7 seconds; Table 1) is below the threshold of 12 seconds to distinguish normal versus below normal mobility [38]. The results are therefore limited in their transferability to more diverse cultural populations with greater mobility restrictions. For pragmatic reasons, we did not include a gold-standard reference measure, that is, optoelectronic measurement, limiting the results of this study accordingly. A segmental analysis of the “up&go app” data (sit-to-stand, walking, turning, and stand-to-sit) was not considered to be useful for self-monitoring. Furthermore, signal noise caused by the pocket-worn approach would be expected, as the smartphone moves back and forth in the trouser pocket in addition to the body movement during the test. Thus, the pocket-worn approach should be considered as a consumer-centered approach and not as a measurement system for segmental or kinematic analysis. For an accurate segmentation of the test, the use of technology that is placed close to the center of mass would be recommended. The instruction to walk at “usual walking speed” leaves room for individual interpretation, hampering standardization. The extent to which the gait speed during the TUG test corresponds to an individual’s actual real-world gait speed was not assessed in this study. It is assumed that performing 5 repetitions and the familiar home setting enabled a greater approximation to the normal walking speed. However, an activity measurement of several days would be required for comparison [51].

### Future Perspectives

The smartphone app used in this study could be suitable as a physical capacity screening and monitoring tool incorporating an instrumented approach for the widely used TUG test. It is designed to be used by older adults, but it could also be implemented by health care professionals to measure TUG total time in clinical settings.

As this study was aimed at validating the test algorithm, it is currently not possible to draw conclusions on the feasibility and user experience of older adults when operating the app, setting up and conducting the test independently. The app should therefore be examined and further developed in future cocreation studies with the target group.

The investigated app is designed to screen physical capacity, representing one of several health domains. In the future, it could be used as part of a comprehensive digital self-assessment, which should include other relevant risk factors such as physical activity, cognition, and vision in addition to physical capacity [24,52]. Self-guided early detection of risks in these domains could enable timely, more specific clinical diagnostics and the initiation of appropriate care interventions. Implementing the app as an upstream assessment within a digital training platform would allow adopting the dosage and supporting the selection of target-specific exercises to be tailored to the user’s individual level of physical capacity.

### Conclusion

The results show excellent concurrent validity and test-retest reliability of the pocket-worn iTUG approach with 5 repetitions. The “up&go app” could be suitable as a self-screening and self-monitoring of physical capacity for older adults at home. It provides a smartphone-based approach to accurately measure the total duration of TUG. This novel approach offers the potential for older adults to take an active role in their health management and preclinical risk detection.

## Abbreviations

ICC: intraclass correlation coefficient
iTUG: instrumented Timed Up and Go Test
LLoA: lower level of agreement
MID: minimal important difference
TUG: Timed Up and Go Test
ULoA: upper level of agreement
UN: United Nations
WHO: World Health Organization
